# Predictors of Psychological Distress and Coronavirus Fears in the First Recovery Phase of the Coronavirus Disease 2019 Pandemic in Germany

**DOI:** 10.3389/fpsyg.2021.678860

**Published:** 2021-12-06

**Authors:** Miriam Biermann, Ruben Vonderlin, Daniela Mier, Michael Witthöft, Josef Bailer

**Affiliations:** ^1^Institute for Psychiatric and Psychosomatic Psychotherapy, Central Institute of Mental Health, Medical Faculty Mannheim/Heidelberg University, Mannheim, Germany; ^2^Department of Clinical Psychology, Central Institute of Mental Health, Medical Faculty Mannheim/University Heidelberg, Heidelberg, Germany; ^3^Department of Psychology, University Konstanz, Konstanz, Germany; ^4^Department of Clinical Psychology, Psychotherapy, and Experimental Psychopathology, Johannes Gutenberg-University Mainz, Mainz, Germany

**Keywords:** COVID-19, corona, pandemics, anxiety, depression, somatic symptoms, health anxiety, information avoidance

## Abstract

**Objectives:** While previous research has mainly focused on the impact of the first acute phase of the COVID-19 pandemic on mental health, little empirical knowledge exists about depression, anxiety, and somatic symptom levels and possible predictors of symptom levels in the pandemic’s recovery phase. The present study aimed to analyze the mental burden of a convenience ample of the general German population during the first recovery phase of the pandemic and to identify significant predictors of symptom levels.

**Methods:** Standardized measures of anxiety (GAD-2), depression (PHQ-2), somatic symptoms (PHQ-15), and health anxiety, as well as measures of COVID-19 fears and possible vulnerability factors, were administered through a national, cross-sectional online survey (*n* = 2160, mean age 42.7 years, 75% female), asking participants for their current symptom levels and their symptom levels prior to the COVID-19 pandemic.

**Results:** Our findings show significantly elevated levels of depression, anxiety, somatic symptoms, and health anxiety in the recovery period compared to before the pandemic. The current prevalence rates based on self-reporting were 26.7% for depression, 24.5% for anxiety, and 29% for somatization. The strongest predictors of these symptom reports included domain-specific pre-existing symptom levels, neuroticism, biological COVID-19 risk factors, avoidance of illness information, and younger age. The most important predictors of COVID-19 fears were subjective COVID-19 risk perception, followed by pre-existing health anxiety, the number of biological COVID-19 risk factors, older age, neuroticism, avoidance of illness information and female gender.

**Discussion:** These findings indicate the need for specific psychological programs to help individuals with enhanced psychological and biological vulnerability to cope better with the mental distress experienced during all phases of the ongoing COVID-19 crisis.

## Introduction

The novel coronavirus (SARS-CoV-2), identified in late 2019 in China (World Health Organization [WHO], 2020b), has rapidly spread worldwide from person to person mainly by respiratory droplets and contact transmission. COVID-19 (**co**rona**vi**rus **d**isease 20**19)** is the infectious disease caused by the novel coronavirus. In the first nine months of the COVID-19 pandemic (until September 27, 2020), more than 32.7 million COVID-19 cases and 991,000 deaths have been reported worldwide to the World Health Organization [WHO], 2020a).

**FIGURE 1:**
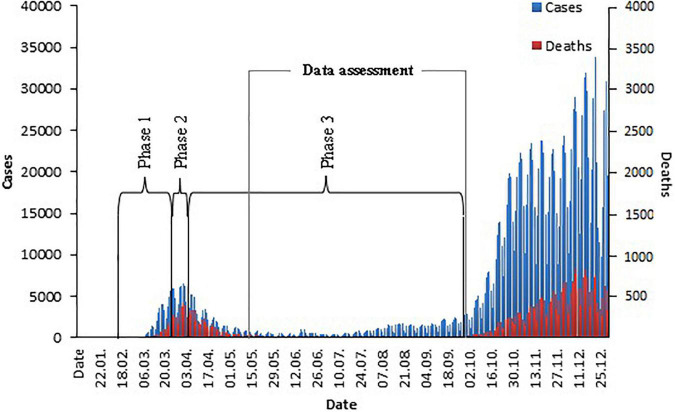
*COVID-19* cases and *COVID-19* related deaths in *Germany* in 2020.

Our current knowledge allows us to divide the current course of the COVID-19 pandemic into three phases (see Figure 1): a preparation phase characterized by a rapid increase of new infections (phase one), the punctum maximum defined by the highest number of new cases (phase two), and a slow return to normality (phase three) (Fegert et al., 2020).

In response to the rapidly rising numbers of COVID-19 cases and deaths in Europe during February and March 2020, many countries implemented large-scale non-pharmaceutical interventions to slow the spread of the coronavirus (including closing preschools, schools, universities, stores, bars, restaurants, hotels, and cultural institutions; stay-at-home policies; border closures; and measures to isolate infected individuals and their contacts). In Germany, the first “lockdown” of public social life started on March 22 and was lifted on April 20. This “lockdown” was effective in reducing virus transmission (Flaxman et al., 2020) and protected the public health system, particularly intensive care units, from a possible breakdown.

These preventive measures, and the economic, social, psychological, and physical consequences of the coronavirus crisis as a whole, however, have had immediate negative effects on people’s mental health and well-being. Several studies carried out during the early phases of the pandemic in China and Europe found increased levels of psychological distress in the general population (Balsamo and Carlucci, 2020; Bäuerle et al., 2020b; Fiorillo et al., 2020; González-Sanguino et al., 2020; Huang and Zhao, 2020; Jia et al., 2020; McCracken et al., 2020; Moccia et al., 2020; Munk et al., 2020; Petzold et al., 2020; Pierce et al., 2020; Ran et al., 2020; Rossi et al., 2020; Shi et al., 2020; Solomou and Constantinidou, 2020; Wang et al., 2020). About one-third of the adult participants in these national surveys was distressed, suffering mainly from generalized anxiety, depression, and perceived stress symptoms. Further, evidence is growing that characteristics such as female gender, younger age, and pre-existing mental health problems were associated with higher distress levels during the early stages of the pandemic (Balsamo and Carlucci, 2020; Bäuerle et al., 2020a,b; Esteban-Gonzalo et al., 2020; Fiorillo et al., 2020; González-Sanguino et al., 2020; Jia et al., 2020; McCracken et al., 2020; Newby et al., 2020; Petzold et al., 2020; Pierce et al., 2020; Rossi et al., 2020; Shi et al., 2020; Solomou and Constantinidou, 2020; Bräscher et al., 2021).

An international systematic review and meta-analysis on 65 longitudinal cohort studies examining changes in mental health among the same group of participants before and during the pandemic found an overall increase in mental health symptoms that was most pronounced during March-April 2020 (Standardized Mean Change, SMC = 0.102 [95% CI:0.026 to 0.192], *p* = 0.03) before significantly declining over time (May-July SMC = 0.067 [95% CI: –0.022 to.157], *p* = 0.141) (Robinson et al., 2021). In addition, results indicate that increases in symptoms of depression and mood disorder tended to be larger (SMC = 0.22, *p* < 0.001) and reductions over time appeared less pronounced as compared with symptoms of anxiety (SMC = 0.13, *p* = 0.02) and general mental health (SMC = –0.03, *p* = 0.65). Studies carried out in Germany found mixed results. One study assessed changes in psychological distress among the general public during the first three months of the pandemic (from March to June 2020) and observed, on average, a weak decrease in psychological distress; however, a subgroup (at least 10% of the respondents) showed an increase in unspecific anxiety and depression symptoms over the time period (Bendau et al., 2020). Another online survey examined the course of psychological distress in the German public from March to April 2020 and observed continuously elevated generalized anxiety scores over time (Hetkamp et al., 2020). Similar results were reported from a representative United Kingdom longitudinal study, with findings showing mental health problems increased from 24.3% before the COVID-19 outbreak to 37.8% in April 2020 and remained elevated in May (34.7%) and June (31.9%) 2020 (Daly et al., 2020).

Since the heterogeneity of the psychological distress associated with the COVID-19 pandemic seems to be considerable, it appears of paramount importance (e.g., for the prevention of psychological distress and allocation of support) to identify the most important correlates and risk factors. In this regard, previous studies suggested that high health anxiety (i.e., the fear of suffering from a severe or life-threatening illness), COVID-19–related media exposure, and neuroticism (i.e., emotional instability) are among the most important factors associated with particularly high levels of psychological distress during the COVID-19 pandemic (Jungmann and Witthöft, 2020; Sauer et al., 2020; Schmidt et al., 2020). Furthermore, additional personality traits according to the BIG-5 model have been found to be significantly associated with COVID-19 anxiety and related general mental distress (Nikčević et al., 2021; Zacher and Rudolph, 2021). Path analytic findings suggest that health anxiety and COVID-19 anxiety serve as significant mediators between personality traits and symptoms of general anxiety and depression, suggesting that both personality traits and health anxiety are important to identify people who are particularly vulnerable to elevated psychological distress associated with the COVID-19 pandemic.

In this context, the purposes of the present study were to (i) investigate how health anxiety and levels of depression, anxiety, and somatic symptoms changed from the time before the rapid spread of COVID-19 (T0) to the first recovery phase (May 12 to September 29, 2020; T1) of the pandemic in Germany, and (ii) determine the predictive value of specific factors associated with symptom levels, health anxiety, and coronavirus fears. We expected enhanced symptom levels and health anxiety during the recovery phase of the pandemic relative to the time before the COVID-19 outbreak. We also expected that specific socio-demographic variables (younger age, female sex, lower education), specific personality traits (i.e., neuroticism), pre-existing levels of health anxiety and illness information avoidance, the number of risk factors linked to a more serious course of COVID-19 (i.e., age > 60 years, smoking, overweight, cardiovascular and other somatic diseases), and perceived risk of infection would predict higher levels of distress, health anxiety, and coronavirus fears during the recovery phase of the pandemic, even when controlling for pre-existing distress levels.

## Materials and Methods

### Design, Recruitment, Participants, and Procedures

A cross-sectional online survey was used to investigate the physical and psychological effects of the coronavirus pandemic in the general population in Germany. We collected data during the recovery phase from the first wave, from May 12 to September 29, 2020, a phase with low numbers of daily new infections and COVID-19–associated deaths. Participants were recruited primarily through press releases (print, online), social media platforms (Twitter, Facebook), the websites at the Central Institute of Mental Health (CIMH), including a COVID-19 mental health support page, and the universities of Mainz and Konstanz. The Ethics Committee of the University of Mainz agreed to conduct the study (2020-JGU-psychEK-S010).

The inclusion criteria of the study were a minimum age of at least 16 years and written informed consent. The exclusion criteria included incomplete processing of the questionnaire and an unrealistically fast total survey completion time (DEG time < 100). In total, 2,224 people started the online survey and 2,160 participants completed it in a realistic processing time. Each participant was asked to report their gender, age, country of birth, highest level of education, employment status, and living situation. We also asked about their experiences with COVID-19 (current or past infection, COVID-19 symptoms, COVID-19–related risk factors, and fears of and perceived risk from COVID-19).

### Measures

To assess somatic symptoms, psychological distress, and health anxiety both for the time before and after the beginning of the COVID-19 pandemic, participants were instructed to answer the same symptom measures twice: first for the current period (T1), then retrospectively for the period before the onset of the pandemic (defined as “the period between the end of February and beginning of March 2020”; T0; this comparatively brief time period was chosen for reasons of standardization and anchor point fixation between participants and in order to use a timeframe that is compatible with the PHQ-4 instruction regarding the previous two weeks).

#### Somatic Distress

Somatic distress was measured using the Patient Health Questionnaire-15, (PHQ-15; Kroenke et al., 2002; Löwe et al., 2002). The PHQ-15 is an excellent and widely used measure of somatic distress and a screening instrument for somatic symptom disorder according to Diagnostic and Statistical Manual of Mental Disorders –5 (DSM-5). The 15 items of the PHQ-15 include the most prevalent somatoform symptoms. The response format consists of a three-point Likert scale ranging from 0 (not at all) to 2 (bothered a lot). The total score ranges from 0 to 30 and scores of ≥ 5, ≥ 10, ≥ 15 represent mild, moderate, and severe levels of somatization, respectively. More importantly, a score ≥ 10 is the most commonly recommended cutoff point for clinically significant symptoms (Kroenke et al., 2010). Internal consistency of the PHQ-15 as assessed with Cronbach’s α was 0.80 in the original validation study (Kroenke et al., 2002) and 0.82 in a large sample, representative of Germany’s general population (Kocalevent et al., 2013). Internal consistencies in our sample were α = 0.84 (T1) and α = 0.82 (T0).

#### Psychological Distress

Psychological distress was assessed using the Patient Health Questionnaire-4 (PHQ-4; Kroenke et al., 2009; Löwe et al., 2010). The PHQ-4 consists of two items assessing core criteria for depressive disorder (little interest or pleasure in doing things; feeling down, depressed, or hopeless) and two items measuring diagnostic criteria of generalized anxiety disorder (feeling nervous anxiety; not able to stop worrying). Participants were asked to indicate how often they have been bothered by these symptoms over the previous two weeks on a four-point Likert scale ranging from 0 (not at all) to 3 (nearly every day). Total scores of the PHQ-4 range from 0 to 12 and scores of ≥ 6 represent at least moderate levels of psychological distress. Internal consistency of the total PHQ-4 as assessed with Cronbach’s α was 0.84 in the original validation study (Kroenke et al., 2009) and 0.82 in a German validation study (Löwe et al., 2010). In the present study, the internal consistencies were α = 0.88 (T1) and α = 0.86 (T0).

#### Health Anxiety and Illness Information Avoidance

Health anxiety was measured using a brief screening instrument specially composed for this study by our working group, the nine-item Health Anxiety Scale (HAS-9; see Supplementary Material 1). All items were taken from well-established health anxiety questionnaires (Barsky et al., 1990; Rief et al., 1998; Salkovskis et al., 2002) based on cognitive-behavioral models of health anxiety and hypochondriasis. The scale covers different facets of the health anxiety construct, such as bodily vigilance (e.g., I am often aware of various things happening within my body), illness-related thoughts and bodily misinterpretations (e.g., Bodily complaints were always a sign of disease for me), and health anxiety (e.g., I am often afraid that I have a serious illness). The statements were answered using a five-point Likert scale ranging from 1 (strongly disagree) to 5 (strongly agree). The total score ranges from 9 to 45, where a higher score indicates higher health anxiety. In the present study, the internal consistencies were α = 0.87 (T1) and α = 0.91 (T0).

In addition, the tendency to avoid illness-related information was measured using three items taken from the avoidance scale of the Questionnaire for Assessing Safety Behavior (QSBH; Weck et al., 2013). All items chosen for the three-item Illness Information Avoidance Scale (IAS-3) refer to the avoidance of illness-related information (i.e., Do you avoid watching documentaries about illnesses? Do you avoid movies or series in which people suffer from a serious illness? Do you avoid reading articles or reports about illnesses?). All items were answered using a five-point Likert scale ranging from 1 (never) to 5 (almost always). The total score ranges from 3 to 15, where a higher score indicates higher avoidance behavior during the previous two weeks. In the present study, the internal consistencies were α = 0.92 (T1) and α = 0.95 (T0).

#### Personality Traits

Personality traits were assessed using the Big Five Inventory-10 (BFI-10; Rammstedt and John, 2007), a short form of the Big Five Inventory (BFI-44; John et al., 1991). The BFI-10 consists of 10 items, based on five factors assessing the big five personality domains: extraversion, neuroticism, openness to experience, conscientiousness, and agreeableness. Each participant indicated how well each statement described their personality on a five-point Likert Scale ranging from 1 (strongly disagree) to 5 (strongly agree). Scores range from 2 to 10 for each of the five personality factors, with higher scores indicating higher levels of the specific personality domain. The BFI-10 has demonstrated good reliability and validity in many samples across different nations (e.g., Rammstedt, 2007; Rammstedt and John, 2007; Carciofo et al., 2016; Kunnel John et al., 2019). Retest-reliability scores at a six-week retest interval were adequate-to-good in the original validation study (Rammstedt and John, 2007).

#### Coronavirus Disease 2019–Related Measures

The survey included several questions regarding COVID-19–related fears, risk perception, and biological risk factors.

##### Coronavirus Disease 2019 Fears

Participants were asked to rate their levels of perceived COVID-19 fear on a visual analog scale ranging from 0 (no fear) to 100 (strong fear) for three different time points: current (item 1), prospectively in four weeks (item 2), and prospectively in eight weeks (item 3) (e.g., How strongly do you fear being infected with coronavirus as of today? How strong do you think your fear of an infection will be four weeks from now?). The mean score of the three items was used as an indicator of perceived coronavirus fear, ranging from 0 to 100. Higher scores indicate higher levels of perceived COVID-19 fears. Cronbach’s α was 0.97.

##### Coronavirus Disease 2019 Risk Perception

Participants were also asked to rate what they thought the likelihood was of being infected with the virus (How likely do you think it is that you will get infected?) or infecting someone else (How likely do you think it is that you will infect someone else?) on a visual analog scale ranging from 0 (very unlikely) to 100 (very likely). The mean score of the two items was used as an indicator of perceived risk of infection, ranging from 0 to 100. Higher scores indicate higher levels of perceived risk of infection. Cronbach’s α was 0.97.

##### Coronavirus Disease 2019 Risk Factors

Participants were asked if they had one or more of the following risk factors (yes, no) for a serious course of COVID-19: higher age (60 years or older), smoking, extremely overweight, cardiovascular diseases (e.g., coronary heart disease or high blood pressure), chronic respiratory disorder (e.g., COPD), chronic liver disease, cancer, or weakened immune system (e.g., due to an illness or regular usage of medicines such as cortisone that lower the immune system). Positive answers were summed up to a risk factor index ranging from 0 to 9, with higher scores indicating higher biological risk for a serious course of COVID-19.

##### Current/Past Coronavirus Disease 2019 Infection

Participants were asked if they were/are known to have been infected with Corona virus in the past or currently. Responses were recorded using a binary variable (yes vs. no).

##### Days After the Peak of the First Coronavirus Disease 2019 Infection Wave

For each participant, the days after the peak of the first wave of infection of Corona were recorded to control for the temporal interval between T1 and T0. The peak of the first wave was set for the 2nd of April, as this was the day with the highest daily incidence in Germany of 6,550 new infections according to the Robert Koch Institute.

### Statistical Analysis

First, we conducted descriptive analyses to describe sample characteristics. Second, we investigated changes in psychological and somatic distress and health anxiety between T0 and T1 using dependent sample *t*-tests for dimensional variables. Third, we conducted chi-square tests for categorical variables to investigate the effects of gender on the prevalence of depression, anxiety, and psychological distress. Fourth, we conducted Pearson correlations to explore associations between predictor and outcome variables. Finally, structural equation modeling was used to explore the independent relationships of predictor variables with outcome variables (current levels of distress as assessed with the PHQ-15 and PHQ-4, health anxiety, and COVID-19 fears). Two of the four outcome variables (PHQ-15 T1 and PHQ-4 T1) were modeled as latent variables. For the PHQ-15-T1 measurement model, a general-factor model with correlated error terms capturing the symptom-specific variance was applied. In case of the PHQ-4, we used a general factor model with correlated error terms between the two anxiety symptoms and the two depression symptoms, respectively. The following 16 predictor variables were entered simultaneously as manifest variables into a latent regression model: pre-existing symptom levels (somatic symptoms, anxiety and depression scores at T0), socio-demographic variables (age, gender, education), personality traits (extraversion, neuroticism, openness, conscientiousness, and agreeableness), pre-existing levels of health anxiety and avoidance behavior (illness information avoidance at T0), and COVID-19 variables (number of COVID-19 risk factors, COVID-19 risk perception, days after the peak of the first COVID-19 infection wave, current/past COVID-19 infection). The analysis was conducted in MPlus Version 7.3 (Muthèn and Muthèn, 2010) using the robust mean and variance adjusted weighted least squares (WLSMV) procedure. Because the chi^2^ test is known for its sensitivity regarding sample size and model complexity, we additionally used common absolute (RMSEA) and comparative (CFI, TLI) fit indices for model fit evaluation. The remaining analyses were performed using SPSS Statistics Version 23 (IBM, Armonk, NY), and the level of significance was set at *p* ≤ 0.01 because even unimportant effects can be significant in large samples. Cohen’s *d* was calculated as effect size for *t*-tests (*d* ≥ 0.30 small effect, *d* ≥ 0.50 medium effect, *d* ≥ 0.80 large effect) (Cohen, 1988). A total of 2,160 participants completed the survey; however, 36 participants had missing values for the “education” variable and 10 participants reported a diverse sex and were excluded, leaving 2,114 participants included in the following analyses. For the predictor analysis, the variables gender and education were dichotomized (gender: female vs. male; education: less than 12 years of schooling vs. more than 12 years of schooling).

## Results

### Sample Characteristics

Of the 2,160 participants who completed the questionnaire, 74.8% were female, 24.7% were male and 0.5% were of diverse sex. The average age was 42.75 years (range: 16–86). Concerning their living situations, 20.9% reported living alone, 71.9% reported living with a partner, family or someone else, and 7.2% reported living in a shared apartment. With regard to education, 78.0% reported having at least 12 years of schooling, and 48.0% had a university degree. With regard to coronavirus, 0.2% said they were currently infected with the coronavirus, and 1.1% said they had been infected with the coronavirus in the past. Thus, the mean infection rate with the Corona virus was 1.3% in our sample compared to 0.24% in the German population (data reported by the Robert Koch Institute on July 21, the median of our survey period). 1.2% reported that a person close to them is currently infected with the coronavirus, and 12.2% reported having a close person who was infected in the past. At least 21.5% of the respondents reported having medium-to-severe fears about coronavirus infection, at least 22.8% expected their fears to remain moderate over the next four weeks, and 23.8% expected their fears to remain moderate to severe over the next eight weeks. At least 30% of the respondents estimated that they were at least 50% likely to become infected with the coronavirus in the future, 31.3% expected with a probability of at least 50% to become a carrier themselves, and 41.4% reported a medium-to-severe fear of becoming a carrier. 19.5% stated that they have been moderately to severely affected by the COVID-19 pandemic in their daily lives. With regard to COVID-19 risk factors, 12.9% were older than 60 years, 18.6% reported that they were smokers, 10.7% stated that they were extremely overweight, 12.7% had cardiovascular disease (e.g., coronary heart disease and hypertension), 5.6% had chronic lung disease [e.g., chronic obstructive pulmonary disease (COPD)], 1.5% had chronic liver disease, 3.0% had diabetes mellitus, 1.8% had cancer, and 7.0% said they had a weakened immune system (e.g., due to illness or regular medication).

### Changes in Psychological and Somatic Symptoms and Health Anxiety

#### Psychological Distress

On average, the current PHQ-4 symptom score (T1), covering anxiety and depression symptoms, was significantly higher than the score calculated for the period before the onset of the coronavirus pandemic (T0) [*M* = 3.56, *SD* = 3.06 vs. *M* = 2.39, *SD* = 2.44; *t*(2113) = 19.81, *p* < 0.001; *d* = 0.42].

#### Somatic Symptoms

Current PHQ-15 mean score (T1) was also significantly higher compared to the score at T0 [*M* = 7.28, *SD* = 5.18 vs. *M* = 5.01, *SD* = 4.17; *t*(2113) = 27.77, *p* < 0.001; *d* = 0.48].

#### Health Anxiety

Finally, the current level of health anxiety (HAS-9 scores at T1) was also significantly higher than at T0 [*M* = 20.51, *SD* = 6.67 vs. *M* = 18.26, *SD* = 6.95; *t*(2113) = 29.51, *p* < 0.001; *d* = 0.33].

The categorization of the participants using established cutoffs (indicating at least moderate expression of symptoms) showed that 10.7% of respondents reported clinically relevant symptoms of depression (PHQ-2 scores ≥ 3), 11.9% reported symptoms of anxiety (GAD-2 scores ≥ 3), 9.4% reported symptoms of psychological distress (PHQ-4 scores ≥ 6), and 12.3% reported symptoms of somatization (PHQ-15 scores ≥ 10) before the outbreak (T0). In contrast, a higher number of participants showed elevated symptom levels during the current phase of the pandemic (T1): the proportion was 26.7% for depression, 24.5% for anxiety, 22.5% for psychological distress, and 29.0% for somatization.

#### Gender Effects

At T0, being female (and not male) was positively associated with the likelihood of probable anxiety [13.0% vs. 8.5%, χ^2^ (1) = 7.65, *p* = 0.006], psychological distress [10.3% vs. 6.5%, χ^2^ (1) = 7.03, *p* = 0.008], and somatization [13.8% vs. 7.8%, χ^2^ (1) = 13.29, *p* < 0.001], whereas no significant gender effect was found for depression [11.3% vs. 9.1%, χ^2^ (1) = 1.95, *p* = 0.163]. At the current phase of the pandemic (T1), being female was positively associated with the likelihood of anxiety [25.6% vs. 21.1%, χ^2^ (1) = 4.49, *p* = 0.034] and somatization [32.3% vs. 19.0%, χ^2^ (1) = 34.25, *p* < 0.001], but not the likelihood of depression [27.3% vs. 24.7%, χ^2^ (1) = 1.45, *p* = 0.228] or psychological distress [23.4% vs. 19.6%, χ^2^ (1) = 2.70, *p* = 0.100].

### Predictors of Distress, Health Anxiety, and Coronavirus Disease 2019 Fears

#### Correlations

In order to investigate predictors of psychological and somatic distress, health anxiety, and COVID-19 fears, first Pearson correlations between the predictor and the outcome variables were computed (see Supplementary Material 2). Inter-correlations of the predictor variables ranged from *r* = 0.00 to *r* = 0.61, with the highest correlation between PHQ-15 (T0) and PHQ-4 (T0). Inter-correlations of the outcome variables ranged from *r* = 0.15 to *r* = 0.64. Again, the highest correlation was found between PHQ-15 (T1) and PHQ-4 (T1), indicating a moderate overlap of these measures. Finally, the correlations between predictor and outcome variables varied between *r* = 0.00 and *r* = 0.82. The great majority of the predictor variables correlated significantly with every outcome variable, with the highest correlation between HAS-9 (T0) and HAS-9 (T1).

#### Results of the Structural Equation Model

The main results of a structural equation model that was used to identify the most relevant predictors of mental distress assessed during the first recovery phase of the pandemic are presented in Table 1. The model fit indices indicate a good model fit according to generally accepted standards (e.g., Schermelleh-Engel et al., 2003).

**TABLE 1:** Identifying predictors of distress, health anxiety and Covid-19 fears severity using a structural equation model (SEM) with WLSV estimation (model fit: CFI = 0.966; TLI = 0.957; RMSEA = 0.030; 90% CI [0.029,0.032]).

	Outcome variables											
	Anxiety and depression			Somatic symptoms			Health anxiety			Covid-19 fears		
	(PHQ-4; T1)^a^			(PHQ-15; T1)^a, b^			(HAS-9; T1)			(CFS-3)		
Predictor Variable	Estimate	SE	p	Estimate	SE	p	Estimate	SE	p	Estimate	SE	p
PHQ-15, T0	–0.010	0.027	0.712	**0.697**	**0.022**	** < 0.001**	0.026	0.016	0.098	–0.011	0.022	0.609
PHQ-4, T0	**0.396**	**0.027**	** < 0.001**	–**0.112**	**0.025**	** < 0.001**	–**0.071**	**0.017**	** < 0.001**	–0.032	0.024	0.174
HAS-9, T0	–0.007	0.024	0.778	0.017	0.022	0.430	**0.781**	**0.010**	** < 0.001**	**0.188**	**0.020**	** < 0.001**
Age	–**0.151**	**0.023**	** < 0.001**	–**0.097**	**0.022**	** < 0.001**	–0.027	0.015	0.065	**0.113**	**0.021**	** < 0.001**
Sex	0.009	0.021	0.655	–0.020	0.021	0.337	**0.045**	**0.013**	**0.001**	–**0.063**	**0.018**	**0.001**
Education	0.000	0.021	0.992	–0.014	0.020	0.475	–0.030	0.012	0.014	0.027	0.018	0.131
BFI-10-Ext	–0.002	0.020	0.906	0.011	0.021	0.604	0.010	0.013	0.450	–**0.064**	**0.018**	** < 0.001**
BFI-10-Neu	**0.271**	**0.022**	** < 0.001**	**0.172**	**0.023**	** < 0.001**	**0.114**	**0.014**	** < 0.001**	**0.101**	**0.020**	** < 0.001**
BFI-10-Ope	0.037	0.020	0.060	0.039	0.020	0.047	0.025	0.012	0.037	0.016	0.017	0.346
BFI-10-Con	–0.003	0.021	0.902	–0.024	0.020	0.219	–0.013	0.013	0.313	0.007	0.017	0.669
BFI-10-Agre	–0.011	0.020	0.595	–0.013	0.020	0.517	0.001	0.013	0.969	–0.025	0.017	0.140
IAS-3, T0	**0.082**	**0.021**	** < 0.001**	**0.077**	**0.021**	** < 0.001**	**0.035**	**0.012**	**0.004**	**0.083**	**0.017**	** < 0.001**
*N* of Covid-19 risk factors	**0.112**	**0.023**	** < 0.001**	**0.116**	**0.021**	** < 0.001**	**0.041**	**0.012**	**0.001**	**0.131**	**0.019**	** < 0.001**
CRPS-2	0.011	0.019	0.575	–0.004	0.020	0.825	0.030	0.012	0.013	**0.487**	**0.014**	** < 0.001**
COVID-9 infection	0.007	0.019	0.715	0.001	0.020	0.957	–0.011	0.020	0.582	–0.041	0.020	0.042
Days after	**0.055**	**0.020**	**0.005**	0.044	0.019	0.024	0.017	0.012	0.157	**0.083**	**0.017**	** < 0.001**

*T0 = retrospective evaluation before the spread of Covid-19; T1 = current evaluation; PHQ-15 = Patient Health Questionnaire 15-Item Somatic Symptom Severity Scale; PHQ-4 = Patient Health Questionnaire 4-Item Anxiety and Depression Symptom Severity Scale; HAS-9 = 9-Item Health Anxiety Scale; Sex = female coded 0 and male coded 1; Education = less than 12 school years coded 0 and 12 or more coded 1; BFI-10-Ext = Big Five Inventory-10-Extraversion; BFI-10-Neu = Big Five Inventory-10-Neuroticism; BFI-10-Ope = Big Five Inventory-10-Openness; BFI-10-Con = Big Five Inventory-10-Conscientiousness; BFI-10-Agre = Big Five Inventory-10- BFI-Agreeableness; IAS-3 = 3-Item Illness Information Avoidance Scale; Covid-19 risk factors = number of risk factors linked to a serious course of Covid-19; CRPS-2 = 2-Item Covid-19 Risk Perception Scale; COVID-9 infection = history of own COVID 19 infection; Days after = time lag (days) between time point of data collection and peak (02.04.2020) of the first wave of the pandemic; ^a^ PHQ-4; T1 and PHQ-15; T1 represent latent variables; ^b^ PHQ-15; T1: the gender specific item 4 (“menstrual cramps or other problems with your periods”) was excluded to avoid gender bias. Bold values represent significance level of p ≤ 0.01; n = 2114.*

##### Psychological Distress (PHQ-4 T1)

Higher pre-existing levels of psychological distress (PHQ-4 T0), higher neuroticism, younger age, a higher number of biological COVID-19 risk factors, and more pronounced avoidance of illness information at T0 were significantly associated with higher levels of psychological distress at T1 (*p* **≤** 0.001 for all). The model accounted for 40.8% of the variance in the latent psychological distress score.

##### Somatic Symptom Distress (PHQ-15 T1)

A similar pattern of significant predictors emerged for current levels of somatic symptom distress. Higher pre-existing levels of somatic symptoms (PHQ-15 T0), higher neuroticism, a higher number of biological COVID-19 risk factors, younger age, and more avoidance of illness information at T0 predicted significantly higher levels of somatic distress at T1 (*p* ≤ 0.001 for all). The model accounted for 59.8% of the variance in the latent somatic symptom score.

##### Health Anxiety (T1)

Again, higher levels of pre-existing health anxiety (T0), higher neuroticism, being male, a higher number of biological COVID-19 risk factors, and more avoidance of illness information at T0 were significant predictors of higher levels of current health anxiety at T1 (*p* **≤** 0.001 for all). The model accounted for 69.3% of the variance in health anxiety scores.

##### Coronavirus Disease 2019 Fears (T1)

The model identified the highest number of significant associations between the predictors and the level of COVID-19 fears (*p* **≤** 0.001 for all). Coronavirus Disease 2019 risk perception, pre-existing health anxiety (T0), number of biological COVID-19 risk factors, being older, neuroticism, avoidance of illness information at T0, number of days after the peak of the first wave, and female gender were all significant predictors of higher COVID-19 fears (all *ps* **≤** 0.001), whereas higher extraversion predicted lower COVID-19 fears. The model accounted for 41.5% of the variance in COVID-19 fears score.

Finally, our results show that current or past coronavirus infection was related to lower COVID-19 fears (*p* = 0.045), but not to symptoms of anxiety and depression, somatization or general health anxiety (all *ps* > 0.50).

## Discussion

### General Discussion of Our Findings

Current research has revealed clear evidence that the “first wave” of the COVID-19 pandemic and the subsequent restrictive measures adopted to slow the spread of the virus are related to increased levels of depression, anxiety, and general distress in different populations around the world. However, changes in these symptom measures over different phases of the pandemic, especially levels of psychological and somatic distress in the recovery period, are heterogeneous. Accordingly, the present study examined the extent of psychological and somatic distress in the recovery period between the first and second waves of the COVID-19 pandemic in Germany. To investigate possible changes in distress levels in the German population, participants answered the same symptom measures twice: first for the current recovery period (T1), then retrospectively for the period before the onset of the COVID-19 pandemic (T0).

In our study, we observed elevated levels of psychological and somatic distress and health anxiety in the recovery period compared to before the pandemic. On average, participants rated their current symptoms of depression, anxiety, somatization, and health anxiety significantly higher than before the onset of the pandemic. Applying established cutoff scores for at least moderate levels of symptoms, approximately 25% of participants experienced psychological and somatic symptoms. Our prevalence rates before the outbreak (T0) are similar to those observed in representative German population samples in the years before the pandemic (Kocalevent et al., 2013; Hajek et al., 2020). In these nationally representative validation studies of the PHQ-4 and PHQ-15 screenings, the prevalence was 10.4% for depression, 9.8% for anxiety, and 9.3% for somatization syndromes. Interestingly, women had higher risk of both anxiety and somatization in these representative samples, which has also been shown in the present study. Our results are based on a convenience sample recruited online who were mostly women (75%) which may explain the slightly higher prevalence of anxiety and somatization at T0 compared to representative samples (Kocalevent et al., 2013; Hajek et al., 2020).

Furthermore, our results agree with findings from a German longitudinal observational study with four stages of online data collection from March 27, the punctum maximum of the first wave (phase two), to June 15, 2020, the beginning of the recovery phase (phase three) (Bendau et al., 2020). The authors observed only a slight decrease in psychological distress (PHQ-4 scores) from March to June with prevalence rates of 31.0% (T1), 25.9% (T2), 22.1% (T3), and 22.6% (T4). Their last assessment interval overlaps with the beginning of our study period and provided nearly identical prevalence rates to those assessed in the following months of the recovery phase in our study (from May to September 2020) using a comparable sample of the German general population. Another online survey that collected data over a 50-day period after the onset of the COVID-19 outbreak in Germany found a similar pattern of results: while COVID-19 fear decreased within six weeks to the level before the lockdown, generalized anxiety remained elevated over time (Hetkamp et al., 2020), indicating no return to the pre-pandemic level.

The study’s second aim was to identify significant predictors of distress and COVID-19 fears during the first recovery phase of the pandemic. As expected, and in line with prior research on the impact of pre-existing mental conditions on current mental health status (e.g., Fiorillo et al., 2020; González-Sanguino et al., 2020; McCracken et al., 2020; Newby et al., 2020), the strongest predictor of current levels of psychological and somatic distress was the domain-specific pre-existing distress level, retrospectively assessed for the period before the onset of the pandemic. Similar strong domain-specific associations were found for current health anxiety, which was best predicted by past health anxiety, and for COVID-19 fears, which were best predicted by higher levels of perceived risk of infection.

Furthermore, younger age was associated with higher psychological and somatic distress. These findings are consistent with previous research on distress during the early phase of the pandemic (e.g., Balsamo and Carlucci, 2020; Bäuerle et al., 2020a; González-Sanguino et al., 2020; Jia et al., 2020; Newby et al., 2020; Nwachukwu et al., 2020; Pierce et al., 2020; Ran et al., 2020; Rossi et al., 2020; Shevlin et al., 2020; Solomou and Constantinidou, 2020). Possible reasons include generally higher social mobility among younger people, little experience with socioeconomic or major life events or pandemics, and higher perceived threat of their academic, social occupational, and economic prospects compared to older people over 25 years (Huang and Zhao, 2020; Wang et al., 2021). As expected, high levels of neuroticism were significantly associated with all outcome measures in this study. Higher neuroticism scores predicted higher levels of psychological distress, somatic distress, health anxiety, and COVID-19 fears, whereas higher extraversion scores were significantly associated with lower levels of COVID-19 fears. These findings are consistent with the vulnerability model, which postulates that neuroticism is an important vulnerability factor for the development of unspecific mental distress and common mental disorders, including anxiety and depression (Jeronimus et al., 2016). In addition, extraversion has shown a robust positive link with subjective psychological well-being in a recent meta-analysis of the links between personality traits and well-being (Anglim et al., 2020).

In line with previous findings from the first acute phase of the pandemic (Jungmann and Witthöft, 2020; Sauer et al., 2020), pre-existing health anxiety predicted current health anxiety and COVID-19 fears but not distress. Moreover, the tendency to avoid illness-related information (e.g., documentaries about illnesses), which is often used as avoidance behavior by health-anxious individuals (Weck et al., 2013), was significantly associated with current health anxiety and with higher levels of current psychological and somatic distress and COVID-19 fears. These findings are consistent with previous research, which has shown that health anxiety, neuroticism, and coronavirus anxiety were significant predictors of depression, generalized anxiety, and death anxiety experienced during the COVID-19 crisis in the United States (Lee et al., 2020). Approximately half (46%) of the current sample surveyed had one or more risk factors to suffer a more severe course of COVID-19. Our results confirm the impact of self-reported biological COVID-19 risk factors (i.e., age > 60 years, smoking, overweight, cardiovascular and other somatic diseases) on psychological and somatic distress and COVID-19 fears. Furthermore, higher subjective risk perception, including the risk of contracting the virus or infecting someone else, was associated with higher COVID-19 fears, but not with higher psychological distress or health anxiety. The number of days after the peak of the first COVID-19 infection wave was also significantly associated with higher COVID-19 fears and elevated levels symptoms of depression and anxiety, which seems consistent against the background of rising infection numbers again in Germany from September onward. Finally, a current or past coronavirus infection predicted lower covid-19 anxiety, which seems plausible against the background of an expected temporary immunization against further Coronavirus infection, as discussed in Germany at that time (Robert Koch Institute, 2021).

### Strengths and Limitations

The major strength of this study relates to the examination of a variety of different variables that predict coronavirus-related psychological distress, somatic symptoms, health anxiety, and COVID-19 fears using structural equation modeling. In addition, to the best of our knowledge, this is one of the first studies to investigate the possible effects of COVID-19 on mental health during the complete recovery phase between the first and the second “waves” in Germany. Nonetheless, several limitations of the present study that may limit the interpretation of our findings must be considered.

The first limitation is that the data at T0 were assessed retrospectively, which might have introduced a memory bias in terms of remembering disorder-specific symptoms, stress and behaviors. There are studies that indicate that retrospective reports of change in mental health are prone to substantial bias during the Corona pandemic (Hipp et al., 2020) and beyond [e.g., (Ben-Zeev et al., 2009; Van den Bergh and Walentynowicz, 2016)]. Despite this substantial limitation of the significance of our findings, the comparability of our retrospective results with cross-sectional prevalence data assessed in the years before the pandemic (Kocalevent et al., 2013) argues against the presence of such a bias. Furthermore, there is evidence that potential bias at the individual survey level is reduced by aggregating data (Jaspers et al., 2009). Additionally, various empirically recommended criteria for ensuring the highest possible reliability and validity of the study’s statements despite retrospective questioning were followed in our study; these include the use of short, easy-to-understand questions, as well as the use of fixed anchor points (for recommendation of retrospective survey questions in COVID-19 studies see Hipp et al., 2020). Furthermore, there are no studies to date that have investigated the validity of our measurement instruments in retrospective use. Due to the sudden spread of COVID-19, it was not possible to implement a longitudinal design with our participants before the outbreak; we decided to adopt an economical solution by applying these partly unvalidated self-report questionnaires. Future studies should incorporate newly published instruments to assess COVID19 related fear (e.g., Ahorsu et al., 2020; Lee et al., 2020; Taylor et al., 2020).

Second, our sample was recruited as a convenience sample mainly through social media and a COVID-19 mental health support page of the CIMH, which might have led to a sample bias. People who have easy access to or are familiar with social media might have been overrepresented in this study. Furthermore, as previous studies indicate (Bendau et al., 2020), people who experience a relatively high level of COVID-19–related psychological distress and who are looking for support might be particularly attracted by social media and might have been more likely to participate in our study. Additionally, the number of men was lower than that of women. This selection bias may partly overestimate the symptom severity and impact of COVID-19, especially given past studies have shown worse impact of pandemics on those with pre-existing mental illness, of younger age and in the female gender (Balsamo and Carlucci, 2020; Bäuerle et al., 2020a; González-Sanguino et al., 2020; Hajek et al., 2020; Jia et al., 2020; Newby et al., 2020; Nwachukwu et al., 2020; Pierce et al., 2020; Ran et al., 2020; Rossi et al., 2020; Shevlin et al., 2020; Solomou and Constantinidou, 2020).

Third, there are several differences between the demographics in our sample and the general population in Germany. Our sample consisted of 74.8% female participants. Since previous studies on the 12-month prevalence of mental disorders in Germany indicate a higher prevalence in women (33% versus 22% in men), the rate and intensity of depressive, anxious, somatization or health anxiety symptoms might be biased (Jacobi et al., 2014). Regarding sociodemographic variables relevant to corona risk, the proportion of participants 60 years and older was lower in our sample than in the general German population [12.7% in our sample versus 29% in the German population; (Statistisches Bundesamt, 2020)]. In contrast, a larger proportion of people than in the general German population were affected by conditions that, according to the Robert Koch Institute, increase the likelihood of corona infection or a severe course [e.g., coronary heart disease; 12.7% in our sample versus 9.3% in the German population; (Gößwald et al., 2013)]. Since our results suggest that younger people but at the same time those with more corona risk factors might be affected by anxiety, depression, health anxiety or somatization symptoms, these differences between our sample and the general German population represent limitations in the generalizability of the findings of this study. In addition, the prevalence rates of corona risk factors were solely based on self-report (e.g., when asked about a weak immune system), which may be subject to social desirability, self-report errors and poor recall and must be considered when interpreting the results. Furthermore, the proportion of people being currently or in the past infected with the Coronavirus was about five times higher among our study participants compared to the German population. Given the Structural Equation Model (SEM) finding that current or past coronavirus infection was associated with lower COVID-19 fears, our results may represent lower levels of COVID-19 fears compared to the general population, limiting the generalizability of the findings.

Finally, although we selected potential predictor variables based on previous studies examining psychological distress under COVID-19, additional important predictors might exist that should be examined in future research.

## Conclusion

In sum, our findings suggest that levels of mental distress were still elevated in this sample of the general German population during this first recovery phase of the pandemic compared to the period before the onset of the pandemic. Women, younger people, those with higher pre-existing levels of distress, higher health anxiety, higher neuroticism, and those with one or more of the known biological COVID-19 risk factors were at higher risk of increased mental distress. Despite the retrospective data assessment and the non-representative sample, our findings provide additional empirical evidence pointing to the need for specific and low-threshold psychological programs to support individuals with enhanced psychological and biological vulnerability to cope with coronavirus-related mental distress during all phases of the ongoing COVID-19 pandemic (Galea et al., 2020; Liu et al., 2020; Vonderlin et al., 2021).

## Data Availability Statement

The raw data supporting the conclusions of this article will be made available by the authors, under limited conditions.

## Ethics Statement

The studies involving human participants were reviewed and approved by the Ethics Committee of the University of Mainz agreed to conduct the study (2020-JGU-psychEK-S010). The patients/participants provided their written informed consent to participate in this study.

## Author Contributions

JB, DM, and MW contributed to the conception and design of the study. JB, MB, and MW organized the database, the acquisition, and performed the data analysis. RV, DM, and MW contributed to interpretation of data for the work. JB and MB wrote the first draft of the manuscript. All authors contributed to manuscript revision, read, and approved the submitted version.

## Conflict of Interest

The authors declare that the research was conducted in the absence of any commercial or financial relationships that could be construed as a potential conflict of interest.

## Publisher’s Note

All claims expressed in this article are solely those of the authors and do not necessarily represent those of their affiliated organizations, or those of the publisher, the editors and the reviewers. Any product that may be evaluated in this article, or claim that may be made by its manufacturer, is not guaranteed or endorsed by the publisher.
