# Supplementary material for: Predictors of Psychological Distress and Coronavirus Fears in the First Recovery Phase of the Coronavirus Disease 2019 Pandemic in Germany
Source: Front Psychol. 2021 Dec 6;12:678860. doi: 10.3389/fpsyg.2021.678860 (PMC8685313; doi:10.3389/fpsyg.2021.678860)
Supplement: Supplementary file 1 [file Table_1.DOCX]

**Supplementary Material 1**

Health Anxiety Scale (HAS-9) English version.

Instruction A: Please indicate how strongly the following statements are applicable to your experience during the past 2 weeks (during the COVID-19 pandemic).

| Source | Ref | Item content | Strongly disagree | Disagree | Neither agree nor disagree | Agree | Strongly agree |
| --- | --- | --- | --- | --- | --- | --- | --- |
| CABAH_14 | (42) | I consult a doctor as soon as possible when I have bodily complaints. | (1) | (2) | (3) | (4) | (5) |
| CABAH_11 |  | Bodily complaints are always a sign of disease. | (1) | (2) | (3) | (4) | (5) |
| CABAH_1 |  | If something is wrong with my bodily sensations, it upsets me at once. | (1) | (2) | (3) | (4) | (5) |
| SSAS_3 | (41) | I am often aware of various things happening within my body. | (1) | (2) | (3) | (4) | (5) |
| HAI_13c | (43) | If I notice an unexplained bodily sensation I often find it difficult to think about other things. | (1) | (2) | (3) | (4) | (5) |
| HAI_3d |  | I am constantly aware of bodily sensations or changes. | (1) | (2) | (3) | (4) | (5) |
| HAI_5c |  | I am often afraid that I have a serious illness. | (1) | (2) | (3) | (4) | (5) |
| HAI_4c |  | I try to resist thoughts of illness but am often unable to do so. | (1) | (2) | (3) | (4) | (5) |
| HAI_7c |  | I often have difficulty in taking my mind off thoughts about my health. | (1) | (2) | (3) | (4) | (5) |

Health Anxiety Scale (HAS-9) English version.

Instruction B: Please indicate how strongly the following statements are applicable to your experience during the past 2 weeks before the COVID-19 pandemic started in Germany (the time between end-February and begin-March 2020).

| Source | Ref | Item content | Strongly disagree | Disagree | Neither agree nor disagree | Agree | Strongly agree |
| --- | --- | --- | --- | --- | --- | --- | --- |
| CABAH_14 | (42) | I consulted a doctor as soon as possible when I had bodily complaints. | (1) | (2) | (3) | (4) | (5) |
| CABAH_11 |  | Bodily complaints were always a sign of disease for me. | (1) | (2) | (3) | (4) | (5) |
| CABAH_1 |  | If something was wrong with my bodily sensations, it upset me at once. | (1) | (2) | (3) | (4) | (5) |
| SSAS_3 | (41) | I was often aware of various things happening within my body. | (1) | (2) | (3) | (4) | (5) |
| HAI_13c | (43) | If I noticed an unexplained bodily sensation I often found it difficult to think about other things. | (1) | (2) | (3) | (4) | (5) |
| HAI_3d |  | I was constantly aware of bodily sensations or changes. | (1) | (2) | (3) | (4) | (5) |
| HAI_5c |  | I was often afraid that I had a serious illness. | (1) | (2) | (3) | (4) | (5) |
| HAI_4c |  | I tried to resist thoughts of illness but was often unable to do so. | (1) | (2) | (3) | (4) | (5) |
| HAI_7c |  | I often had difficulty in taking my mind off thoughts about my health. | (1) | (2) | (3) | (4) | (5) |
