# Supplementary material for: Predictors of Psychological Distress and Coronavirus Fears in the First Recovery Phase of the Coronavirus Disease 2019 Pandemic in Germany
Source: Front Psychol. 2021 Dec 6;12:678860. doi: 10.3389/fpsyg.2021.678860 (PMC8685313; doi:10.3389/fpsyg.2021.678860)
Supplement: Supplementary file 2 [file Table_2.DOCX]

**Supplementary Material 2**

Table 1: *Pearson correlations within and between predictor and outcome variables*

|  | *Predictor variables* | | | | | | | | | | | | | | | | *Outcome variables* | | | |
| --- | --- | --- | --- | --- | --- | --- | --- | --- | --- | --- | --- | --- | --- | --- | --- | --- | --- | --- | --- | --- |
|  | 1 | 2 | 3 | 4 | 5 | 6 | 7 | 8 | 9 | 10 | 11 | 12 | 13 | 14 | 15 | 16 | 17 | 18 | 19 | 20 |
| 1 PHQ-15 T0 | 1 |  |  |  |  |  |  |  |  |  |  |  |  |  |  |  |  |  |  |  |
| 2 PHQ-4 T0 | **0.61** | 1 |  |  |  |  |  |  |  |  |  |  |  |  |  |  |  |  |  |  |
| 3 HAS-9 T0 | **0.41** | **0.43** | 1 |  |  |  |  |  |  |  |  |  |  |  |  |  |  |  |  |  |
| 4 IAS-3 T0 | **0.16** | **0.15** | **0.34** | 1 |  |  |  |  |  |  |  |  |  |  |  |  |  |  |  |  |
| 5 BFI-10-E | **-0.15** | **-0.22** | **-0.13** | **-0.08** | 1 |  |  |  |  |  |  |  |  |  |  |  |  |  |  |  |
| 6 BFI-10-N | **0.33** | **0.47** | **0.31** | **0.11** | **-0.27** | 1 |  |  |  |  |  |  |  |  |  |  |  |  |  |  |
| 7 BFI-10-O | 0.03 | 0.03 | **0.06** | -0.01 | **0.13** | **-0.06** | 1 |  |  |  |  |  |  |  |  |  |  |  |  |  |
| 8 BFI-10-C | **-0.14** | **-0.20** | **-0.13** | -0.01 | **0.22** | **-0.16** | **0.11** | 1 |  |  |  |  |  |  |  |  |  |  |  |  |
| 9 BFI-10-A | **-0.11** | **-0.12** | **-0.13** | -0.04 | **0.15** | **-0.14** | **0.07** | **0.12** | 1 |  |  |  |  |  |  |  |  |  |  |  |
| 10 CRFI | **0.20** | **0.08** | **0.09** | **0.06** | -0.02 | -0.02 | -0.02 | **-0.08** | -0.05 | 1 |  |  |  |  |  |  |  |  |  |  |
| 11 CRPS-2 | **0.19** | **0.18** | **0.21** | 0.03 | **-0.08** | **0.16** | 0.05 | **-0.08** | -0.01 | -0.03 | 1 |  |  |  |  |  |  |  |  |  |
| 12 Age | **-0.08** | **-0.19** | -0.03 | **0.17** | 0.01 | **-0.16** | 0.01 | **0.14** | -0.05 | **0.40** | **-0.20** | 1 |  |  |  |  |  |  |  |  |
| 13 Sex | **-0.16** | **-0.08** | -0.03 | -0.02 | -0.06 | **-0.18** | **-0.08** | **-0.12** | **-0.09** | **0.10** | **-0.07** | **0.13** | 1 |  |  |  |  |  |  |  |
| 14 Education | **-0.10** | **-0.06** | -0.04 | -0.05 | -0.02 | -0.02 | **0.14** | 0.04 | 0.01 | **-0.18** | 0.06 | **-0.10** | -0.01 | 1 |  |  |  |  |  |  |
| 15 Days after | 0.00 | -0.02 | 0.05 | **0.08** | -0.03 | -0.01 | **-0.08** | 0.04 | **-0.13** | **0.08** | -0.04 | **0.18** | 0.04 | **-0.07** | 1 |  |  |  |  |  |
| 16 COV-Inf | 0.00 | -0.01 | -0.03 | -0.03 | 0.02 | -0.05 | -0.01 | -0.05 | 0.04 | 0.02 | -0.02 | **-0.06** | **0.06** | 0.05 | 0.02 | 1 |  |  |  |  |
| 17 PHQ-15 T1 | **0.70** | **0.45** | **0.32** | **0.16** | **-0.12** | **0.36** | 0.03 | **-0.13** | **-0.09** | **0.17** | **0.16** | **-0.13** | **-0.17** | **-0.08** | 0.03 | 0.01 | 1 |  |  |  |
| 18 PHQ-4 T1 | **0.34** | **0.53** | **0.27** | **0.14** | **-0.16** | **0.44** | 0.03 | **-0.14** | **-0.10** | **0.09** | **0.13** | **-0.18** | -0.06 | -0.04 | 0.03 | 0.00 | **0.64** | 1 |  |  |
| 19 HAS-9 T1 | **0.36** | **0.35** | **0.82** | **0.31** | **-0.12** | **0.34** | 0.06 | **-0.13** | **-0.12** | **0.11** | **0.21** | -0.03 | -0.00 | -**0.07** | **0.06** | -0.03 | **0.45** | **0.38** | 1 |  |
| 20 CFS-3 | **0.22** | **0.20** | **0.36** | **0.21** | **-0.16** | **0.24** | 0.03 | -**0.06** | **-0.09** | **0.17** | **0.52** | **0.07** | **-0.09** | 0.01 | **0.11** | **-0.07** | **0.20** | **0.15** | **0.38** | 1 |

*Note.* T0 = retrospective evaluation before spread of Covid-19; T1 = current evaluation; PHQ-15 = Patient Health Questionnaire Somatic Symptom Severity Scale; PHQ-4 = Patient Health Questionnaire Anxiety and Depression Symptom Severity Scale; *CFS-3* = COVID-19 Fears Scale*.* HAS-9 = 9-Item Health Anxiety Scale; IAS-3 = 3-Item Illness Information Avoidance Scale; BFI-10-E = Big Five Inventory-10-Extraversion; BFI-10-N = Big Five Inventory-10-Neuroticism; BFI-10-O = Big Five Inventory-10-Openness; BFI-10-C = Big Five Inventory-10-Conscientiousness; BFI-10-A = Big Five Inventory-10- BFI-Agreeableness; CRFI = COVID-19 risk factors index, e.g. number of risk factors linked to a serious course of COVID-19; CRPS-2 = 2-Item Covid-19 Risk Perception Scale. Sex = female coded 0, male coded 1; Education = less than 12 school years coded 0 and 12 or more coded 1; Days after = time lag (days) between time point of data collection and peak (02.04.2020) of the first wave of the pandemic. COV-Inf = Current/past COVID-19 infection. Bold values represent signicance level of *p* ≤ .01; *n* = 2114.
